# Efficacy and safety of fibrin sealant application in patients undergoing thyroidectomy: a systematic review and meta-analysis

**DOI:** 10.1186/s12893-024-02414-2

**Published:** 2024-04-24

**Authors:** XiaoGang Zheng, Fan Wang, Yong Cheng Su, Chao Yang Xu, Ming Zheng Wang

**Affiliations:** 1 Jinhua Maternity and Child Health Care Hospital, Surgery, Xia man University, Jinhua City, 324100 China; 2 Jinhua Central Hospital, Surgery, Zhejiang University, Jinhua City, 324100 China; 3 Xiamen Key Laboratory for Tumor Metastasis, Cancer Research Center, School of Medicine, Xiamen University, Xiamen, 361102 China; 4 Jinhua Central Hospital, Surgery, Shandong University, Jinhua City, 324100 China

**Keywords:** Thyroid surgery, Fibrin sealants, Wound drainage, Length of hospitalization, Meta-analysis

## Abstract

Various studies have focused on the application of fibrin sealants (FS) in thyroid surgery. Utilizing a meta-analysis, this systematic review analyzed the findings of recent randomized controlled trials on the safety and efficacy of FS in patients who underwent thyroidectomy. The Cochrane Library, Web of Science, Embase, PubMed, and Medline databases were searched for relevant studies, without any language restrictions. Seven randomized controlled trials were included in the originally identified 69 studies. Overall, 652 patients received FS during thyroid surgery; their outcomes were compared with those of conventionally treated patients. The primary outcomes were total volume of wound drainage, length of hospitalization, and operative time. Significant differences were observed in the total volume of wound drainage (mean deviation (MD): -29.75, 95% confidence interval (CI): -55.39 to -4.11, *P* = 0.02), length of hospitalization (MD: -0.84, 95% CI: -1.02 to -0.66, *P* < 0.00001), and surgery duration (MD: -7.60, 95% CI: -14.75 to -0.45, *P* = 0.04). Secondary outcomes were seroma and hypoparathyroidism development. The risk of hypoparathyroidism did not differ between the FS and conventional groups (I = 0%, relative risk = 1.31, *P* = 0.38). Analysis of “seroma formation that required invasive treatment” indicated that FS showed some benefit (I^2^ = 8%, relative risk 0.44, *P* = 0.15). Heterogeneity among the different trials limited their conclusions. The meta-analysis showed that although FS use did not significantly reduce seroma or hypoparathyroidism incidence in patients after thyroidectomy, it significantly reduced the total drainage volume, length of hospitalization, and duration of surgery.

## Introduction

Surgery is the most common treatment option for thyroid pathologies [1]. However, this type of intervention is not free from complications such as postoperative hematoma, hypoparathyroidism, delayed drain removal, and seroma, all of which can lead to patient discomfort. The most serious postoperative complication is hematoma, which can be life-threatening. The incidence of hemorrhage after thyroidectomy ranges from 0.5 to 4.3% [2], whereas seroma, an accumulation of inflammation-associated exudate following surgery, occurs during the acute wound-healing stage [3]. Seroma occurs in approximately 14% of conventionally performed thyroidectomies, resulting in difficulty swallowing and neck pain due to compression [4]. Improved approaches such as ultrasound cutting devices [5], meticulous dissection of the thyroid gland, suction drainage systems [6], and the application of fibrin sealants (FS) [7] have been developed to overcome the above-mentioned complications. However, suction drainage systems induce inflammation and may also aggravate drainage, as the negative pressure of the drain causes a vacuum that prevents the sealing of the lymphatics and hence increases the likelihood of drainage and seroma formation [8].

FS comprise high concentrations of fibrinogens mixed with other cryoglobulins, which often appear in the form of patches or glues [9]; for example, the FS made from fibrinogen and thrombin that are used to improve wound healing. FS are commercially available, and several extensively studied products have been approved by the US Food and Drug Administration (FDA) for use in different areas of surgery. FS are widely used in head and neck surgery, where they have been found to decrease wound drainage and contribute to improved short-term recovery following surgery [10]. The drainage output after thyroidectomy is reduced by the fibrin glue [11], which may be associated with reduced postoperative bleeding. Although the effects of hemostatic agents have been extensively investigated in other fields, the impact of FS use during thyroid surgery remains unclear [10, 12]. The design and performance of several types have been criticized, whereas others have yielded inconclusive results [13]. Here, a systematic review and meta-analysis of randomized controlled trials (RCTs) of patients who underwent thyroid surgery were conducted to evaluate the efficacy and safety of FS.

## Materials and methods

A review protocol was developed and used to guide this meta-analysis. The Preferred Reporting Items for Systematic Reviews and Meta-Analyses (PRISMA) [14] guidelines were followed.

### Information sources and search strategy

The Embase, PubMed, Web of Science, MEDLINE, and Cochrane Library online databases were selected to search and retrieve electronically available literature published between July 1980 and July 2022. The search terms used were “Fibrin adhesive tissue,” “fibrin glue,” “fibrin sealant,” “thyroid gland,” “thyroidectomy” and “thyroid neoplasms.” In addition, unpublished studies in English were searched on the ClinicalTrials.gov registry (https://clinicalinicaltrials.gov/) using a “randomized controlled trial.” This systematic review was performed in accordance with PROSPERO online database guidelines (CRD42022344496).

### Eligibility criteria

The selected studies conformed to the following criteria: (i) those in which conventional thyroid surgery and surgery using FS were compared; (ii) those in which the outcomes of factors such as age, length of hospitalization, transient hypocalcemia, operative time, drainage output, and seroma and hematoma formation were reported; (iii) RCTs; and (iv) studies in which at least one measure of variance was provided (confidence interval, standard deviation, or standard error). In cases where a similar study resulted in more than one publication, only the most informative study was included. Finally, the authors of the original papers were contacted when data were difficult to determine. Meeting abstracts and full-text articles were included to avoid potential publication biases.

### Data extraction and quality assessment

The primary outcomes were “volume of drainage,” “length of hospital stay,” and “operative time” operative time. The secondary outcomes were “hypoparathyroidism” and “seroma”. A standardized data collection table was constructed and used independently by two experienced authors (Z.X.G and W.F) to extract the following information from eligible studies [15]: name of the first author, year and country of publication, study design, ethnicity, surgical methods, planned sample size, number of randomizations, and number of dropouts. The opinion of a third reviewer was used to settle disputes [16]. The Cochrane Collaboration tool for bias risk assessment was used for the analysis of study methodology and bias risk, addressing the randomization procedures, concealment of allocation, blinding, outcomes, follow-up, and intention-to-treat [17].

### Statistical analysis

RevMan version 5.4.1 (Cochrane Collaboration) and Stata 15.0 were used for data analysis. The statistical heterogeneity of the studies was assessed using standard chi-square tests (statistical heterogeneity was considered at a significance level of *P* < 0.1) and evaluated using the I^2^ statistic [18]. Differences in means for continuous and dichotomous outcomes in each trial and differences in means were calculated using 95% confidence intervals (CI). The effects from individual trials were further combined using a random-effects inverse variance weighted method for continuous outcomes, while the Mantel-Haenszel method was used for dichotomous outcomes. Furthermore, I^2^ statistics and sensitivity analysis were used to assess the degree of heterogeneity under the assumption of fixed treatment effects to enable comparisons. It was further assumed that presenting continuous data as medians and interquartile ranges (IQRs) would result in skewed information; hence, the authors of the manuscripts were asked to provide actual means and standard deviations (SDs). However, if this was unsuccessful and there was a minimal degree of bias, the mean and SD were estimated for the meta-analysis while accepting the limitations of this approach [19]. The means and SDs were also estimated if no response was received from the authors of the studies, where data were presented as medians with minimum and maximum values [20]. *P*-values < 0.05 were considered significant for all statistical analyses [21].

## Results

### Study selection

**Fig. 1:**
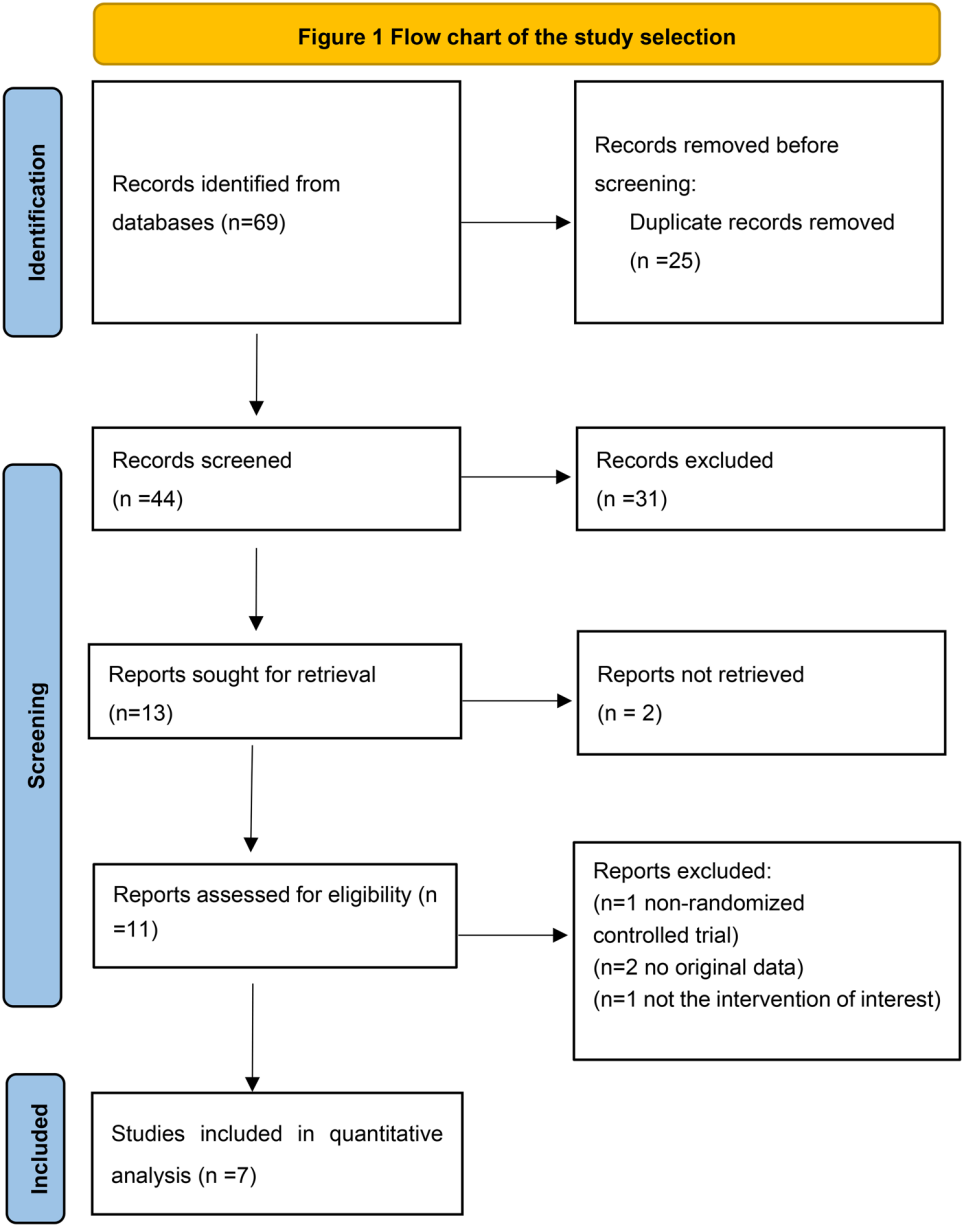
PRISMA diagram showing the process of study selection

Figure 1 shows a PRISMA flowchart summarizing the study selection process. After the preliminary selection of 69 studies that did not contain any gray references, 62 studies were excluded based on the inclusion criteria after reviewing their titles and abstracts. Finally, seven RCTs, published between 1980 and 2022 were included, all of which highlighted the importance of FS in thyroid surgery in terms of hemostatic efficiency and safety. Overall, both the FS and control groups contained 326 participants.

The demographic characteristics and patient details are presented in Table 1. Two of the seven studies focused on the application of FS in thyroidectomy, both total and hemithyroidectomy [11, 22], and included total thyroidectomy using some form of neck dissection. More specifically, the trial by Vidal-Perez et al. included patients who had central and lateral neck dissections, while the study by Kim et al. involved patients with central neck dissections [12, 23].

**Table 1:** Details of the included articles

Study	Surgery	Year	Race	Planned sample size	Number randomized	Case (T/C, n)	No. of drop-outs	Intervention	Control
Uwiera et al.	Thyroidectomy	2005	Canada	-	56	26/30	0	Tisseel FS	No FS
S Sözen et al.	Thyroidectomy	2011	Turkey	100	100	50/50	0	CryoSeal FS	With suction drain
Kim et al.	Thyroidectomy and central neck dissection	2012	South Korea	72	78	38/40	0	Berplast P FS	No FS
Hornig et al.	Thyroidectomy	2016	USA	110	70	28/27	15	Evicel FS	Saline Placebo
Vidal-Pérez et al.	Thyroidectomy and neck dissection	2016	Spain	60	60	30/30	0	Tissucol FS	No FS
Geraci et al.	Thyroidectomy	2019	Italy	262	262	134/128	0	Tisseel FS	With suction drain
Eun Ju Ha et al.	Thyroidectomy and neck dissection	2022	South Korea	41	41	20/21	0	6 mL tisseel FS	2 mL tisseel FS

T: treatment group; C: control group; FS: Fibrin sealant

**Fig. 2:**
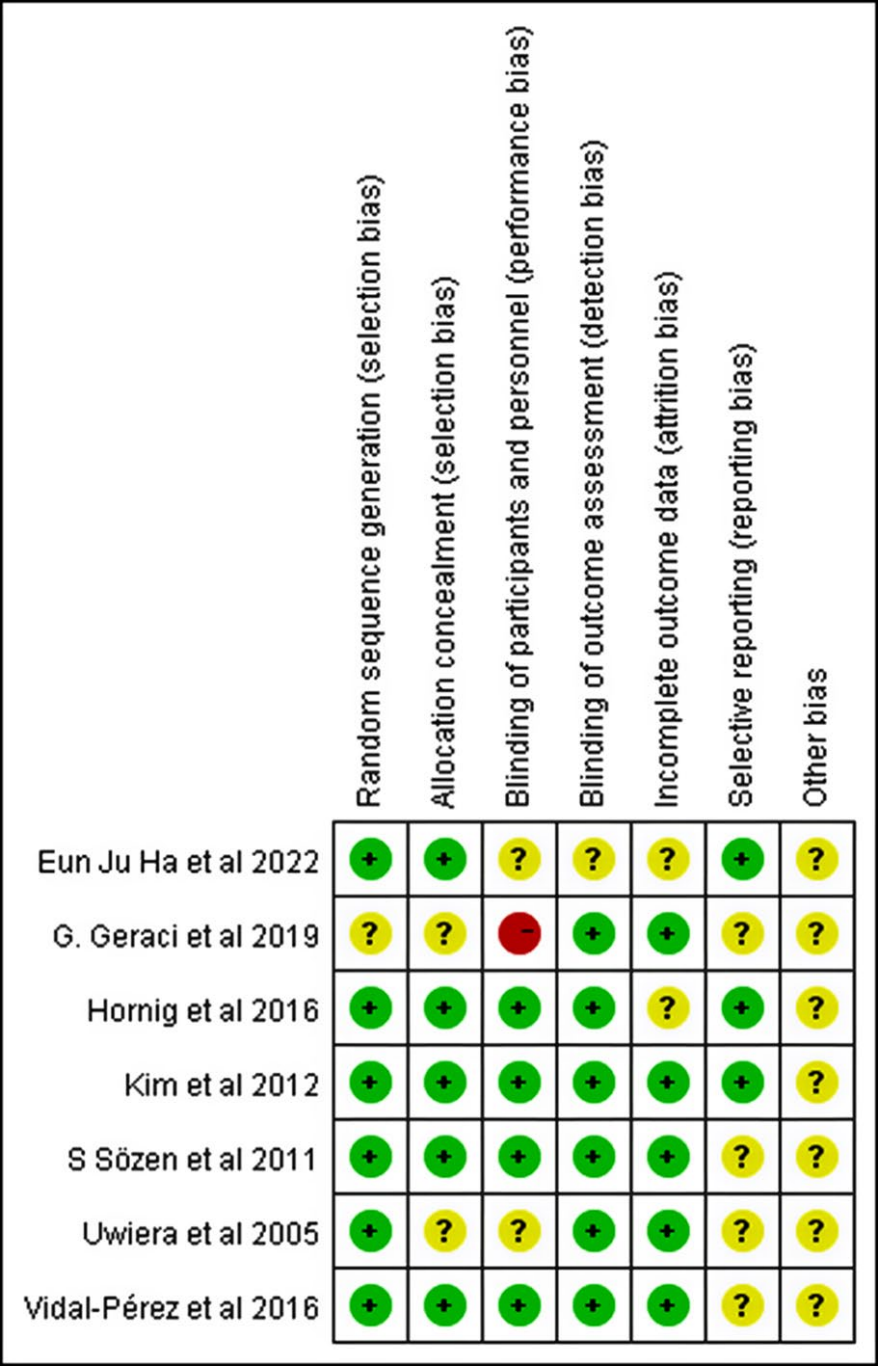
Risk of bias in the included randomized controlled trials (RCTs) on FS application in patients undergoing thyroidectomy. The Cochrane Collaboration risk-of-bias evaluation technique was used to examine the probability of bias in the seven included RCTs using seven criteria [22]. Green, low risk of bias; yellow, uncertainty; red, high risk of bias

Figure 2 shows the assessed risk of bias. Variations were noted in the overall quality of the studies, with many studies providing incomplete methodological data that hindered the ability to fully assess the risk of bias. Despite contacting the authors via email, only two responses were received. The essential finding was that while random sequence generation was mostly adequate, the lack of sufficient selective reporting limited attempts to reduce the selection bias. Furthermore, three studies pre-registered the trial protocols on ClinicalTrials.gov and CRIS to compare the planned and reported outcomes and assess the reporting bias.

### Primary outcomes

#### The total volume of drainage

**Fig. 3:**
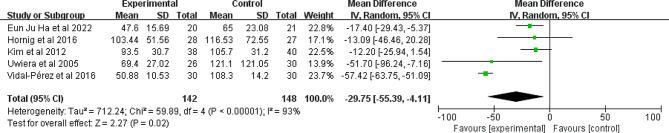
Forest plot showing the effect of FS application on the total wound drainage volume. FS: fibrin sealant

The total drainage volume was provided in five of the trials (Fig. 3), with 142 of 290 patients treated with FS. The FS-based treatment significantly decreased the drainage volume (I^2^ = 93%, MD: -29.75, 95% CI: -55.39 to -4.11, *P* = 0.02).

#### Operative time

**Fig. 4:**
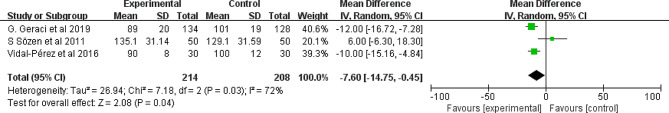
Forest plot of the effect of FS application on operative time. FS: fibrin sealant

Three studies compared the conventional approaches and the use of FS in terms of differences in operative time (Fig. 4). In this case, the application of FS significantly decreased the operative time (I^2^ = 72%, MD: -7.60, 95% CI: -14.75 to -0.45, *P* = 0.04).

#### Length of hospitalization

**Fig. 5:**
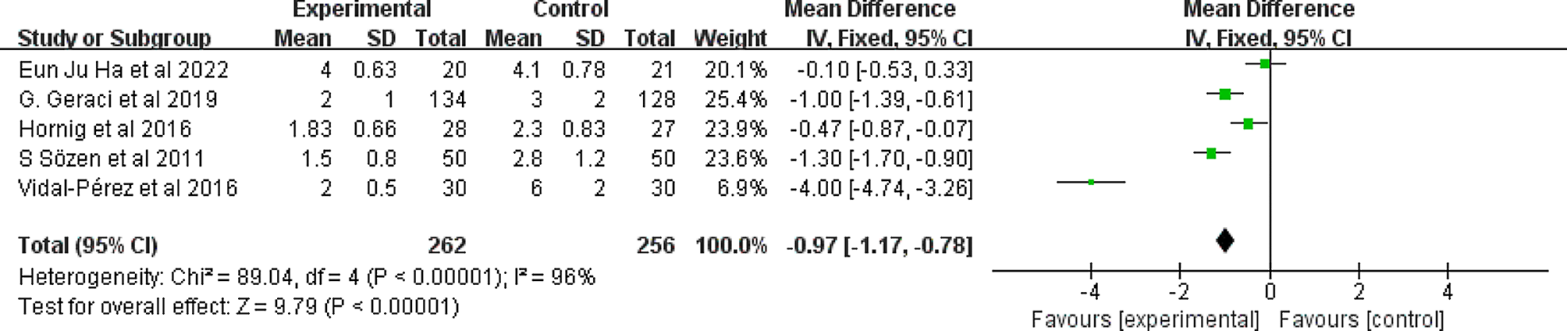
Forest plot showing the effect of FS application on length of hospital stay. FS: fibrin sealant

Figure 5 shows the analysis of the “length of hospitalization” in days. This showed that the use of FS reduced hospital stays by 1.33 days (I^2^ = 96%, 95% CI -2.27, -0.40, *P* = 0.005); however, the results displayed significant statistical heterogeneity.

### Secondary outcomes

#### Seroma

**Fig. 6:**
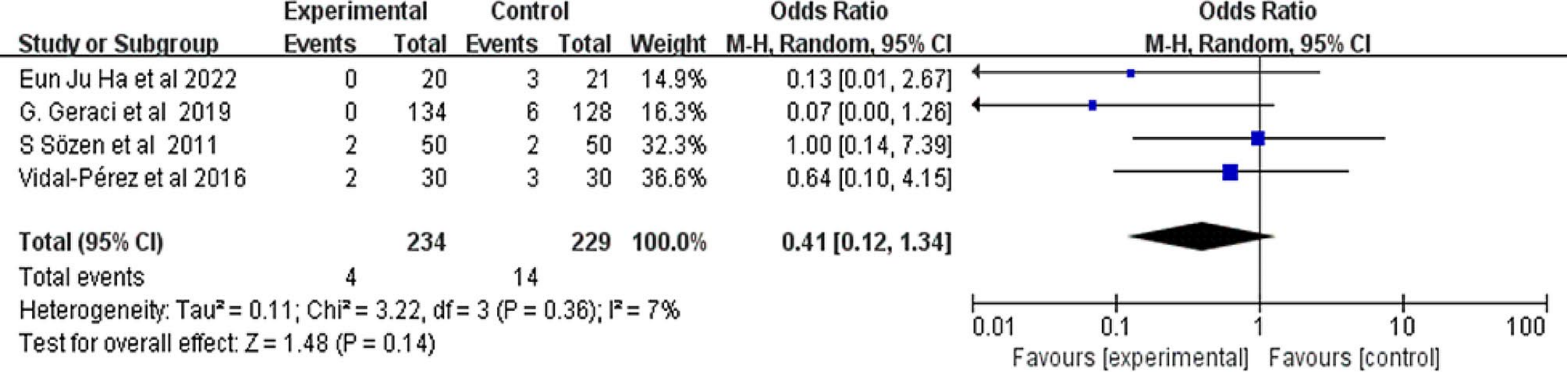
Forest plot showing the effect of FS application on seromas. FS: fibrin sealant

The effects of conventional methods and FS on seromas were determined in four studies (Fig. 6). No significant difference was found between the two approaches (I^2^ = 8%, 95% CI 0.14 to 1.37, *P* = 0.15).

#### Hypoparathyroidism

**Fig. 7:**
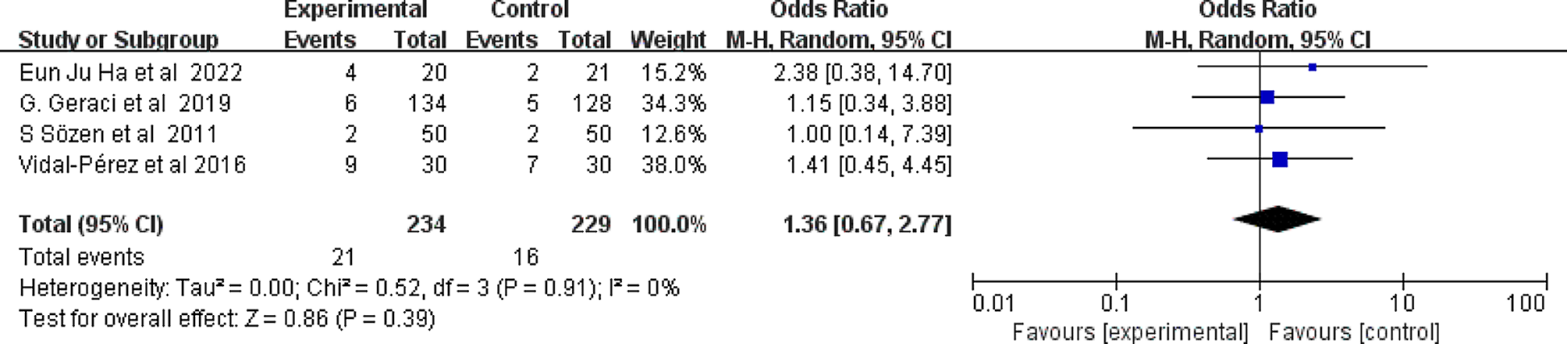
Forest plot showing the effect of FS application on hypoparathyroidism. FS: fibrin sealant

Similarly, the effects of conventional methods and FS on hypoparathyroidism were compared in four studies (Fig. 7). The approaches did not differ significantly in terms of the risk of hypoparathyroidism (I^2^ = 0%, 95% CI 0.72 to 2.37, *P* = 0.38).

### Subgroup analysis

**Fig. 8:**
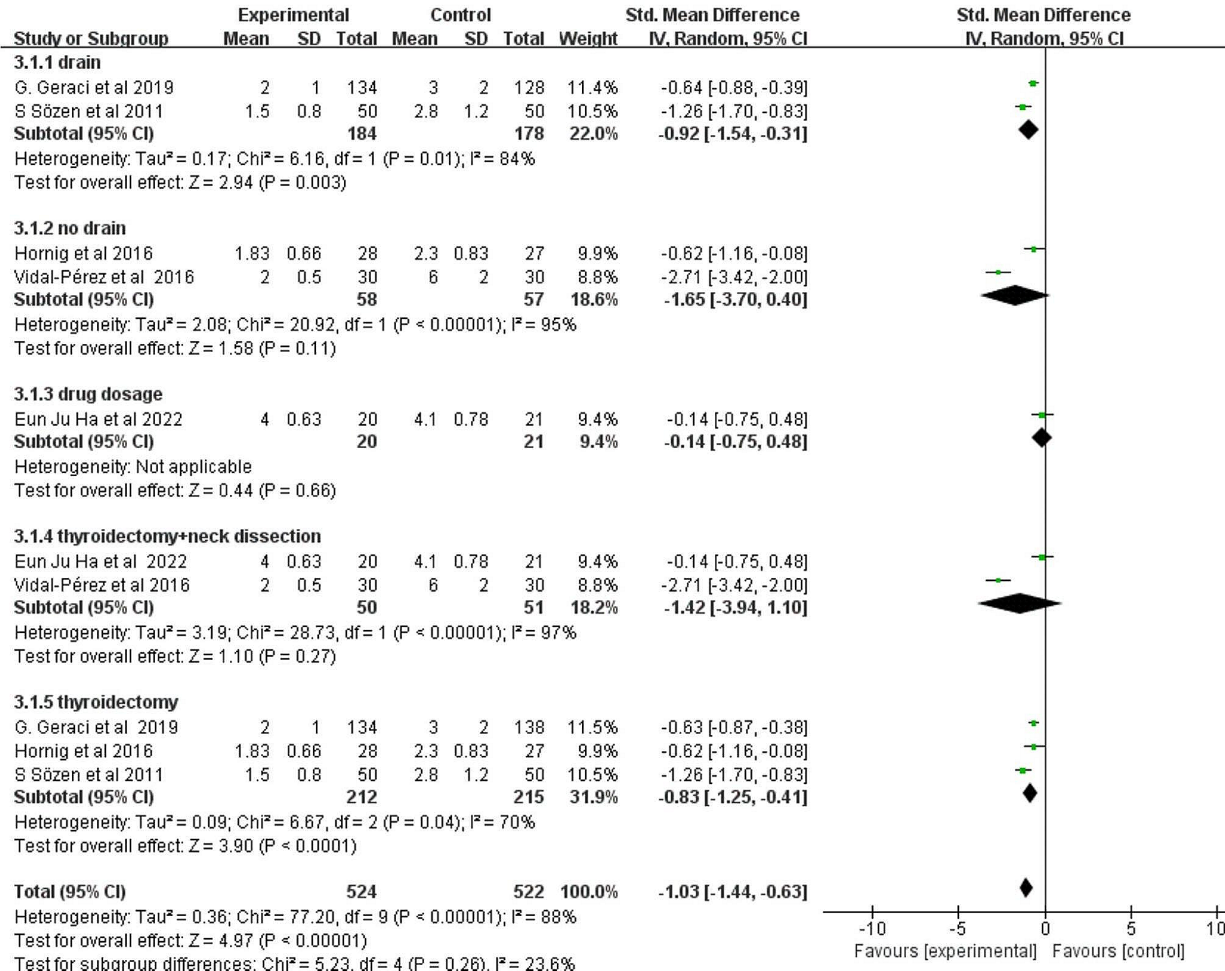
Subgroup analysis of hospital stay

A subgroup analysis was conducted for the length of hospitalization, it was found that the positive effect still existed regardless of whether the drainage was included or the surgical method was changed. The volume of fibrin glue used ranged from 2 mL to 6 mL. Finally, owing to the small number of trials, predefined subgroup analyses aimed at determining whether effect sizes were dependent on FS dosage were uninformative (Fig. 8).

### Sensitivity analysis

**Fig. 9:**
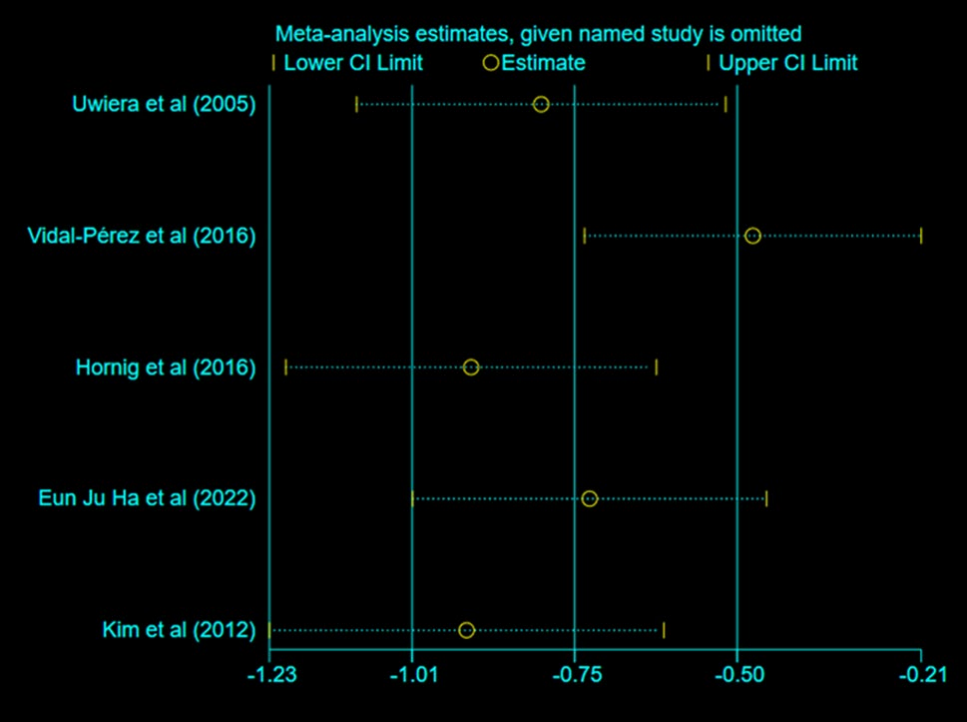
Sensitivity analyses of the included studies in terms of the total wound drainage volume. The two vertical axes represent the 95% CI, and the horizontal axis represents the overall HR. In the current review, the pooled OR was represented by a hollow, round shape, and was excluded from the remaining studies. The two ends of each broken line represent the corresponding 95% CI. CI: confidence interval, HR: hazards ratio, OR: odds ratio

Sensitivity analysis was also performed to estimate the validity of the statistical tests [24]. Except for the data extracted from the study by Vidal-Pérez et al., all affected the combined effect size but did not change the effects of FS application on the total wound drainage volume. The deletion of the study had a small effect on the combined effect value of the remaining studies, confirming the stability of the final results of this analysis (Fig. 9).

## Discussion

Because the thyroid has an abundant vascular supply, intraoperative hemostasis is essential to prevent major consequences. Thyroidectomy can cause various postoperative complications, such as hypoparathyroidism, infections, and seromas, which not only increase morbidity and mortality but also lead to increased drainage and extended recovery times and hospital stays [25]. This review includes a systematic meta-analysis of the efficacy and safety of FS in patients undergoing thyroidectomy. Overall, although FS did not markedly reduce the incidence of seroma, hypoparathyroidism, or other related complications, it did significantly reduce the length of hospital stay and drainage output.

Considerable heterogeneity was observed among the selected RCTs, which may be attributed to the fact that these trials included different surgical procedures. First, the types of thyroid surgery differed, involving either thyroidectomy alone or thyroidectomy accompanied by lateral or central neck dissection. Hornig et al. investigated total thyroidectomy [22], Kim et al. assessed total thyroidectomy with central neck dissection [12], and Vidal-Perez et al. investigated total thyroidectomy with both central and lateral neck dissection [23]. FS may be more beneficial in patients who undergo lateral or central neck dissection. Surgery for malignant diseases tends to be associated with neck dissection, resulting in a much wider postoperative dead space than thyroidectomy alone. Neck dissection is usually associated with significant exposure of the muscles surrounding large vessels and often results in complications owing to its proximity to the airway [26]. Variations in the operative field may have contributed to the observed differences in the drainage volume and seroma formation. Second, there were variations in the formulation or brand of FS used, which could potentially result in different wound-healing efficacies. Finally, the methodologies used to assess the duration of the hospital stay, seroma formation, total volume, and hypoparathyroidism were not uniform.

Regarding “Efficacy”, the advantages of FS have been recognized for many years, and FS have been used in many surgical procedures. For instance, FS reduces the drainage volume and duration of surgery for melanoma (30). Fibrin was also found to markedly reduce drainage volume in patients undergoing surgery for gynecological cancers (31). The application of FS not only enhances angiogenesis and hemostasis to induce wound healing but the FS are also completely absorbed without triggering reactions that normally occur in response to foreign bodies [27]. Theoretically, FS can reduce the accumulation of postoperative serosanguinous fluid and prevent seroma formation by enhancing tissue adherence and hemostasis [4]. Thus, FS application provides significant clinical advantages, such as a reduced incidence of complications (e.g., hematoma or seroma formation and infections) or even reduced drainage of surgical wounds, thereby reducing or even eliminating the need for surgical drains [26].

Regarding “Safety,” there have been several reports of side effects associated with FS, although these have a low incidence. Geraci et al. reported a case of temporary bilateral recurrent stupor that was treated conservatively and was resolved by functional resection and integrum after 32 days. This was most likely due to spraying too close to the surgical site (< 10 cm), leading to tracheoesophageal barotrauma [28].

The meta-analysis further demonstrated that FS could decrease drainage output. However, several studies have reported that the incidence of postoperative bleeding following thyroid surgery is less dependent on the application of intraoperative hemostatic agents than on surgeon skill [14, 18, 21]. However, the authors suggest that FS is useful in conjunction with good surgical techniques. Furthermore, reduced drainage duration may mitigate postoperative discomfort in patients [26]. Thus, FS appears to be an effective adjunct for hemostatic control during thyroidectomy. FS was found to reduce the drainage output, length of hospitalization, and operative time during thyroidectomy.

Throughout this study, a thorough process encompassing a pre-registered review protocol, duplicate screening, data collection, a highly inclusive search strategy, and evaluation of bias risk was implemented [29]. Nevertheless, this study has some limitations. Subgroup analyses were restricted by the reported data, although the surgical procedure addressed some of the variations. Furthermore, the risk of bias, which affects most of the included RCTs, limits the quality of evidence. The efficacy of the sealant may depend on fibrinogen concentration, with low concentrations likely being less effective [30]. Although one study used FS with different fibrinogen concentrations in the included RCTs, the outcomes could not be analyzed because of insufficient data [31]. Moore et al. reported that FS significantly increases the risk of seroma [32]. Some of the results were equivocal. For example, the difference in operative time was 10 min in two studies in favor of FS, but in a third study, the operative time was 6 min faster with no FS. Although this latter study had twice as many patients as one of the other studies, it only had 20% weighting. Further research is required to determine whether FS are safe and effective. In addition, none of the trials reported significant differences between conventional methods and FS in terms of infection.

## Conclusion

Taken together, these results demonstrate that FS are not effective in reducing the incidence of seroma or hypoparathyroidism in patients after thyroidectomy, although they reduce the total drainage volume, length of hospitalization, and operative time. Overall, FS have some benefits in improving the postoperative condition of these patients.

## Data Availability

No datasets were generated or analysed during the current study.
